# Multi-Scale Modeling and Simulation of Thermoplastic Automated Tape Placement: Effects of Metallic Particles Reinforcement on Part Consolidation

**DOI:** 10.3390/nano9050695

**Published:** 2019-05-04

**Authors:** Angel Leon, Marta Perez, Anaïs Barasinski, Emmanuelle Abisset-Chavanne, Brigitte Defoort, Francisco Chinesta

**Affiliations:** 1 ESI Group, 3bis rue Saarinen, 94528 Rungis CEDEX, France; angel.leon@imdea.org (A.L.); Marta.PerezMiguel@esi-group.com (M.P.); 2 GeM, Ecole Centrale Nantes, 1 rue de la Noe, 44300 Nantes, France; anais.barasinski@ec-nantes.fr; 3 I2M, ENSAM ParisTech, Esplanade des Arts et Métiers, 33405 Talence CEDEX, France; emmanuelle.abisset@ensam.eu; 4 ArianeGroup, Rue du Général Niox, BP30056, 33166 Saint-Médard-en-Jalles, France; brigitte.defoort@ariane.group; 5 PIMM Lab & ESI Group Chair, ENSAM Paristech, 151 Boulevard de l’Hôpital, 75013 Paris, France

**Keywords:** reinforced resins, microwires, consolidation, prepreg, squeeze flow, PGD, wavelet surface representation

## Abstract

This paper concerns engineered composites integrating metallic particles to enhance thermal and electrical properties. However, these properties are strongly dependent on the forming process itself that determines the particle distribution and orientation. At the same time, the resulting enhanced thermal properties affect the reinforced resin viscosity whose flow is involved in the intimate contact evolution. Thus, a subtle and intricate coupling appears, and the process cannot be defined by ignoring it. In this paper, we analyze the effects of particle concentration and orientation on the process and processability. For this purpose, three main models are combined: (i) a multi-scale surface representation and its evolution, by using an appropriate numerical model; (ii) flow-induced orientation, and (iii) the impact of the orientation state on the homogenized thermal conductivity.

## 1. Introduction

Over the last decades, an increasing number of functional and structural parts, made so far with metals, have been progressively reengineered by replacing metallic materials by polymers, reinforced polymers, and composites. The motivation for this substitution may be the weight reduction, the simpler, cheaper, or faster forming process, or the ability to exploit additional functionalities [1].

The fillers usually employed cover a broad range involving many scales: (i) the nanometer scale (e.g., carbon nanotubes, graphene, fullerene, nanodiamonds); (ii) the micrometer to the millimeter scale (particles and short fibers); (iii) the centimeter scale of fibers used in SMCs (sheet moulding compounds) and BMCs (bulk moulding compound) composite processes; and finally (iv) the macroscopic scale where fibrous reinforcements are made of continuous fibers arranged in bundles. When load-bearing capacities are especially looked for, continuous fiber reinforced polymers are selected. In that case, the impregnation of the reinforcement with a low viscosity polymer involves the flow of a Newtonian or non-Newtonian fluid in the complex multi-scale microstructure related to the fiber and tow arrangement.

Reinforced polymers are selected instead of high-performance polymers of equivalent properties since the latter are generally more expensive. When looking for functional properties, the use of micro- and nano-particles opens a wide spectrum of possibilities but is also at the origin of new challenges, such as dispersion of particles into the polymer matrix, the occurrence of aggregation and disaggregation mechanisms, and flow-induced particle distribution and orientation, both affecting strongly the final properties of the resulting engineered materials and then the ones associated to the manufactured composite structural parts.

Many composite forming processes for manufacturing structural parts are based on the consolidation of preimpregnated preforms, e.g., sheets and tapes. Among the numerous technological solutions available, automated tape placement (ATP) is increasing its popularity because it allows for avoiding the use of an autoclave with in-situ consolidation.

In ATP processes, a tape is placed and progressively bonded to the substrate consisting of tapes previously laid-up. The cohesion of two thermoplastic layers requires specific physical conditions: intimate contact and a temperature that is high enough to enable molecular diffusion but low enough for avoiding thermal degradation. The last two conditions acting in opposite directions on the heating process define the process window [2].

**Figure 1:**
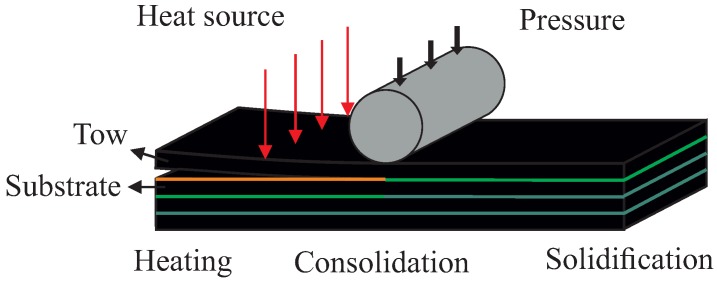
Sketch of the ATP process.

In all cases, due to the low thermal conductivity of usual resins, an intense local heating is usually considered (laser, gas torches, etc.) in conjunction with a local pressure applied by the consolidation roller moving with the heating head, as illustrated in Figure 1. Intense research focuses on consolidation enhancement. In [3], a deep sensitivity analysis was carried out to evaluate the influence of different materials and process parameters of the consolidation, whereas [4] analyzed the influence of the tape surface roughness and concluded that a parameter playing a major role is the surface curvature (evaluated at the roughness level). However, the effects of using metallic particles reinforced resins has not been, in our knowledge, evaluated. We come back to this issue later.

As experiments at very fine scales require specific testing devices, and the determination of sensitivities is a tricky issue, numerical modeling, by using advanced state-of-the-art models and advanced simulation techniques, is an appealing route to ensure a first understanding that then can and should be validated by appropriate experiments.

The numerical model of ATP process was addressed in [5,6] by using advanced numerical techniques, the so-called proper generalized decomposition (PGD) [7]. The separated representation involved in the PGD enables the 3D high-resolution solution of models defined in degenerated domains where at least one of their characteristic dimensions remains much smaller than the others [8] and also constructing solutions of parametric models where the model parameters are considered as extra-coordinates [7].

Many authors have addressed asperity squeezing induced by applied external pressure, considering different representations of surface roughness [9]. The description of random surfaces and the evaluation of roughness on physics occurring at the surface neighborhood was addressed in several works. Fractals were widely considered when addressing self-affinity through space scales [10]. In our recent work, we addressed asperity squeezing considering both linear and nonlinear fluid constitutive equations, and instead of using rectangular representations of asperities we considered a fractal surface representation, taking into account the roughness anisotropy that pre-impregnated composites exhibit [11,12,13]. However, fractal representations were unable to explain experimental findings; in particular, surfaces having the same fractal parameters exhibited different behaviors during consolidation. This issue comes directly from the definition of fractal parameters, which are not sufficient to define unequivocally a surface from the point of view of consolidation purposes. This may result in two different surfaces having the same fractal parameters, surfaces that under the same process conditions exhibit in the end different consolidation degrees. To alleviate these limitations, a description of the real surface based on a wavelet-based multiresolution approximation was proposed in [14], where squeezing flow enables the quantification of consolidation.

Nowadays, the design and use of engineered functional materials is attracting the interest of researchers, who are discovering unexpected phenomena and envisaging new applications. Thus, incorporating metallic particles into resins is improving thermal and electrical conduction. However, composite materials do not exist intrinsically; they become the processing output, and the strong coupling between process, materials, and properties cannot be ignored.

The process affects the reinforcement distribution as well as its orientation, and from both the final material and part properties will follow. On the other hand, the fact of adding particles to the resin will affect the forming itself due to the resin viscosity increase, so a coupled modeling becomes crucial to evaluate the couple material-process.

Different questions arise whose responses are far from being obvious. One of them concerns the effect of particles on the thermal conductivity that is expected to increase. In fact, such an increase was one of the reasons for considering the addition of particles, but, it is not clear how such an increase will affect the process. It is important to note that thermal conductivity is of major relevance with respect to the temperature field evolution all along the process and that such evolution strongly affects the resin viscosity that governs the microscopic and macroscopic flows during consolidation. Moreover, as proved in our former works referred above, the flow not only affects the distribution and orientation of charges; it also affects the evolution of the intimate contact between the incoming ply and the substrate. This intimate contact has a first-order effect on both heat conduction and residual porosity.

Discovering the effects of all these intimate and intricate physics and phenomena by experimental campaigns and the design of appropriate tests seems costly, expensive, and tricky. Using well-established state-of-the-art models, with their associated advanced high-fidelity simulations, allows for the reduction of the need for experimental tests and offers a first model-based understanding, and experiments are reduced to model calibration and solution validation. Moreover, simulation allows isolating “properly” a factor in order to evaluate its impact in the process, with all the other factors kept unchanged. Such a sensitivity analysis remains a tricky issue when addressed experimentally.

The present work is a part of that big picture. It will focus on the numerical analysis of the impact of particles on the process and on the processability, trying to better understand the main phenomena, the key factors, and so on in order to drive validation experiments and model calibration tests. Thus, the present work constitutes the first step towards a deep understanding that should help engineers to better design processes, materials, and structural/functional parts and to define key experiments that validate the proposed approaches and designs. Here, the use of nanometric particles is analyzed. Particles are hundreds of nanometers in diameter and hundreds of micrometers in length, ensuring very large aspect ratios, of the order of a hundred. Different advantages of using these nano-particles are envisaged: (i) the possibility of placing them into a very concentrated medium, where the reinforcement based on carbon fibers is larger of 60%, inducing intense confinement effects; (ii) the possibility to place them at the roughness level (surface asperities); (iii) making possible percolation at a low concentration due to a very large particle aspect ratio; and (iv) profiting from the superior induced properties of nanometric particles with respect to larger size particles.

Section 2 addresses the main methods related to the surface, orientation, and thermal conductivity quantification and evolution. The integration of these three components to the integrated process modeling will be described in Section 3. Section 4 addresses a numerical study to conclude on the effects of particle concentration and the prepreg surface roughness on consolidation.

## 2. Methods

This section proposes a modeling framework for evaluating the evolution of the pre-impregnated tape surface during the in-situ consolidation. It involves the simulation of the squeeze flow and the evaluation of the flow-induced anisotropy of the particle orientation to finally calculate the effective homogenized thermal conductivity.

### 2.1. Surface Description and Its Time Evolution

In [14] a Haar wavelets representation of the surface was proposed, consisting of a sequence of rectangles of different heights and widths. A representative rectangular element was then squeezed under the usual lubrication theory assumptions. The interested reader can refer to that work, which addressed the flow of such a rectangular element for both Newtonian and power law fluids. From this elementary flow solution, a simulation of the set of rectangles approximating the real surface using a Haar wavelet description was performed.

For that purpose, different rectangles representing the surface (Haar wavelet representation) were squeezed. Those in contact with the compression plate flow reduced their heights during displaced fluid flows towards the neighboring rectangular elements. When the neighboring elements, or one of them, are not yet subjected to the effect of compression, their height increases because of the flow they are receiving. When the neighboring elements are also subjected to compression effects, the flow incoming circulates to the following elements. The mass conservation in combination with the moment balance leads to the pressure equation that governs those flows occurring during compression. In that modeling framework, the pressure field in the rectangular elements that are not squeezed by the compression plate vanishes. For a more detailed description, the interested reader can refer to [14].

Compression can be ensured by considering a given compression rate or a compression force. In the former case, roughness squeezing continues until complete roughness removal at a constant rate. In the latter case, the situation is different, because the larger the contact surface is, the higher the applied pressure for ensuring the material displacement at a given rate should be. If the applied force remains constant, the compression rate decreases and perfect contact becomes in most of cases unattainable. Moreover, in this case, if a thermal coupling is considered, a temperature decrease induces a viscosity increase, a fact that results in an almost negligible compression rate. In the coupled case, motion and heat equations are solved, and from the calculated temperature the viscosity is updated, before solving again the motion and heat equation with the just-updated viscosity.

Thus, when the compression only applies during a short time interval, as is the case in usual ATP processes, prescribing the compression force results in residual porosity at the surface level because of the impossibility of squeezing until complete roughness removal.

### 2.2. Particle Orientation Model

The flow-induced orientation of a particle, assimilated to an ellipsoid whose orientation is defined by the unit vector p, can be modeled [15] by using the Jeffery equation [16], given its rotary velocity:

(1) $$\dot{p}=\Omega \cdot p+k\left(D\cdot p-D:\left(p⊗p\right)p\right),$$

where Ω and D are respectively the vorticity and the rate of strain tensors, and k depends on the ellipsoid aspect ratio *r*.

When considering metallic microwires, the aspect ratio can be assumed to be infinite [17] and consequently k≈1, leading to

(2) $$\dot{p}=\nabla v\cdot p-D:\left(p⊗p\right)p.$$

However, the above equation is not sufficient to describe a population of particles in a fluid since it refers to a single particle. In that case, a mesoscopic description introduces the probability distribution function (pdf) $\Psi \left(x,t,p\right)$ describing the fraction of particles that at position x, and time *t* are oriented along direction p. The probability balance results in the so-called Fokker–Planck equation that governs the pdf time evolution:

(3) $$\frac{\partial \Psi }{\partial t}+\nabla_{p}\left(\dot{p}\Psi \right)=0$$

where $\frac{\partial }{\partial t}$ is the material derivative, and $\nabla_{p}$ the gradient operator in the conformation space p. The main drawbacks in using this formalism is its multidimensionality. Thus, coarser descriptions are preferred consisting of the pdf moments, and in particular the second order moment that defines the so-called second order orientation tensor:

(4) $$a\left(x,t\right)=\int_{S}p⊗p\;\Psi \left(x,t,p\right)dp.$$

The time derivative of Equation (4), using Equation (1), leads to the equation governing the orientation tensor evolution

(5) $$\dot{a}=\Omega \cdot a-a\cdot \Omega +k\left(D\cdot a+a\cdot D-2A:D\right),$$

where A is the so-called fourth order orientation tensor defined by

(6) $$A\left(x,t\right)=\int_{S}p⊗p⊗p⊗p\;\Psi \left(x,t,p\right)\;dp,$$

which requires the use of an appropriate closure relation. Here, the quadratic closure relation [18] is considered; when assuming k≈1, it reads

(7) $$\dot{a}=\nabla v\cdot a+a{\left(\nabla v\right)}^{T}-2A:\nabla v.$$

The computer implementation of the flow-induced orientation when compressing a rectangular element has been analyzed in depth in [12].

#### Homogenized Thermal Conductivity

Because the fluid is reinforced by micro-metallic particles, the thermal properties of the reinforced resin change during compression since the orientation of the particles is also affected, which is necessary to determine the change in thermal properties during the compression. There are other works that models the influence of micro-fibers on different material properties [19,20].

To alleviate the computational cost, an effective homogenized thermal conductivity is calculated in a representative volume with particle concentration ϕ and orientation $\Psi \left(p\right)$.

The resin thermal conductivity is assumed to be isotropic and is expressed by $K_{m}$ where the bulk isotropic conductivity of the rod particles are named by $K_{p}$. The heat flux in the matrix is given by the standard Fourier law for a given temperature gradient:

(8) $$Q_{m}=-K_{m}\left(1-\phi \right)\nabla T.$$

In the case of the particles, the heat flux is considered in the direction of the rod direction (it is an appropriate hypothesis taking into account its aspect ratio), so the gradient along the rod is

(9) $${\hat{Q}}_{p}\left(p\right)=-K_{p}\left(p⊗p\right)\cdot \nabla T.$$

Therefore, the heat flux in the rods reads

(10) $$Q_{p}=\int_{\Omega }{\hat{Q}}_{p}\left(p\right)\phi \Psi \left(p\right)dp=-K_{p}\phi a\nabla T.$$

Thus, the total heat flux results of $Q_{c}=Q_{m}+Q_{p}$ are

(11) $$Q_{c}=-\left(K_{m}\left(1-\phi \right)I+K_{p}\phi a\right)\cdot \nabla T$$

where I is the identity matrix. Therefore, the homogenized anisotropic conductivity reads

(12) $$K_{c}=K_{m}\left(1-\phi \right)I+K_{p}\phi a.$$

The computer implementation of the flow-induced effective thermal conductivity when compressing a rectangular element was studied in [12].

## 3. Process Modeling and Simulation

**Figure 2:**
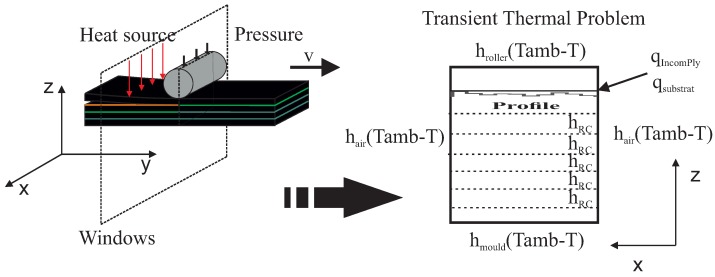
Computational domain.

**Figure 3:**
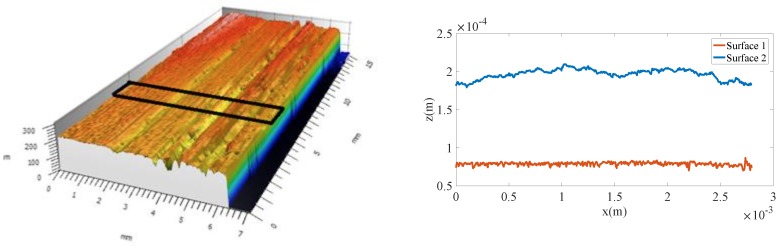
Surface acquisition using a profilometer (**left**) and measured profile (**right**) representing the relative height z(m).

**Figure 4:**
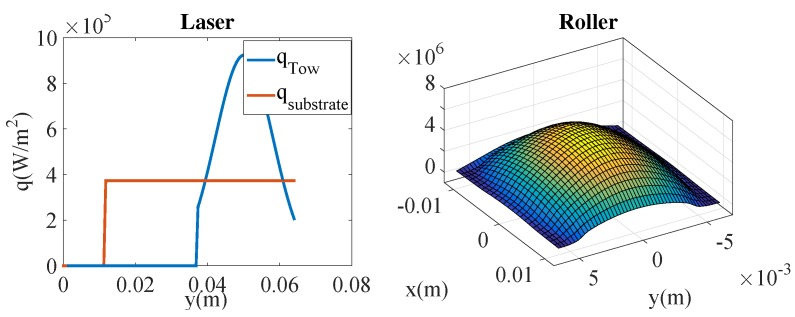
(**left**) Heating flux; (**right**) pressure field applied by the roller associated with a compression force of 600 N (the dimensions being 3 cm (fiber direction) × 1.5 cm (tape width)) − Value of Max Pressure: 5 MPa).

This section aims at analyzing the effect of the different material and process parameters on consolidation. For that purpose, we consider the techniques just described with the hypotheses that follow:

1. An Eulerian frame, in which the tape moves while the laying head remains at rest, is considered. This frame choice allows one to reduce the thermal and squeezing flow calculation to the tape cross-section depicted in Figure 2.
2. The domain of the study consists of an m-plies laminate. We consider more than 6 plies in order to limit the effect of the boundary condition between the substrate and the tool.
3. The tape surface is represented by a sequence of rectangular elements (Haar wavelet representation) constructed from the data provided by a profilometer. Two different prepregs are considered, both of them exhibiting different roughness, as reported in Table 1 and illustrated in Figure 3.
4. The metallic particles contained in the prepreg are considered well dispersed and isotropically oriented at the beginning of the forming stage. The orientation evolution and induced properties are computed all along the consolidation process.
5. Room temperature was assumed as the initial temperature of the incoming tape.
6. Heating was provided by a laser source applied at the tape/substrate interface, with the heat power repartition shown in Figure 4 [21];
7. The pressure applied by the consolidation roller has a spatial distribution depicted in Figure 4, showing an applied force of 600 N [21].
8. Parameters involved in the simulation are reported in Table 2.

## 4. Results

In this study, two different unidirectional prepregs are considered, whose profiles are represented in Figure 3. Both of them involve the same material, PEEK matrix (34%wf) and carbon fiber reinforcement, but are elaborated using different processes of impregnation, the latter having a strong influence on final surface properties, in particular the micro-roughness reported in Table 1.

In order to study the influence of the particle content on the consolidation evolution, we considered different concentrations ϕ, around 1% to avoid an impact on mechanical performance. More precisely, we considered concentrations ranging from 0% to 2%, the needed amount for reaching a percolated regime being about 1% [23,24].

As previously discussed, particles are expected to affect substantially the resin viscosity (the effective viscosity is assumed depending on the particle concentration according to [25]) and its anisotropic homogenized thermal conductivity, both playing a major role in the consolidation process as discussed in Section 1. There are without a doubt other consequences of the particles’ presence, such as the laser/matter interaction or its impact on the resin density and on its specific heat, but they are, in a first approximation, assumed to be of second order and are not considered in the present study.

The process integrating the three main phenomena (surface, orientation, and thermal conductivity evolution), assuming an almost isotropic orientation of the particles immersed in the resin at the initial time, can be simulated. To better differentiate the impact of the viscosity and the thermal conductivity, both of them, indissociable experimentally, are dissociated numerically, in order to analyze the impact of viscosity (without any change in the conductivity) and then the impact of the thermal conductivity for a given viscosity. Of course, such uncoupled behavior is unrealistic but highly valuable from the point of view of a deep process understanding.

**Figure 5:**
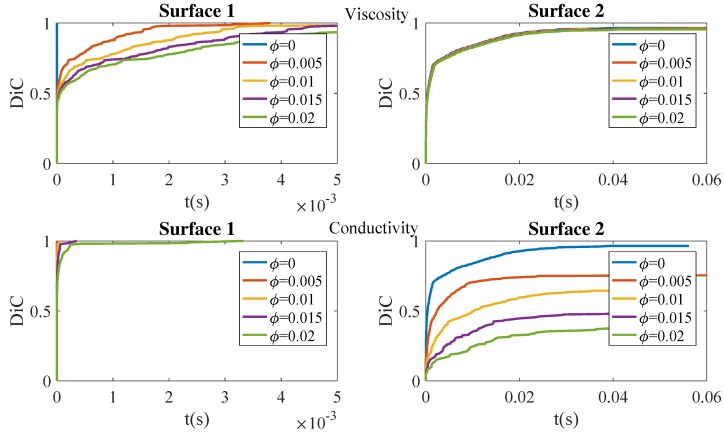
Effect of the filler concentration ϕ on viscosity (**top**) and conductivity (**bottom**): the uncoupled model.

Figure 5 depicts the simulation findings when varying the particle content (ϕ). It can be noticed that the smoothest prepreg surface (Surface 1) reaches very quickly (in about $10^{-3}$ s) an almost perfect consolidation (i.e., a degree of intimate contact (DiC) approaching one), whereas for the roughest surface (Surface 2) an almost perfect consolidation needs about $10^{-2}$ s to $10^{-1}$ s, one or two orders of magnitude more. In the case of the smoothest surface, the impact of the viscosity increase seems more prejudicial than the impact on the thermal conductivity changes. On the other hand, in the case of the roughest one, the increase of the viscosity has a slight impact on the consolidation, the effect of conductivity being of major relevance. For a higher filler content, perfect consolidation is never reached due to the overly intense heat conduction that produces a quick cooling and with it the increase in the viscosity, making the squeezing flow progression difficult.

**Figure 6:**
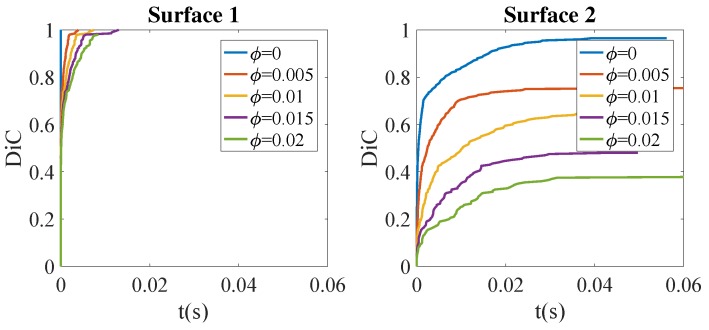
Effect of filler concentration ϕ on the degree of intimate contact: the coupled model.

Figure 6 shows the consolidation evolution when both phenomena are taking place in a coupled manner; that is, fillers affect both the resin viscosity and the thermal conductivity. Surface 1 seems quite insensible to the presence of particles, whereas Surface 2’s processability seems compromised.

## 5. Conclusions

The present paper aims at evaluating the impact of fillers, with an interest to recover or enhance functional properties, on process and processability. Three main physical mechanics where retained in the ATP process modelling: the surface evolution, the charge orientation evolution, and its impact on the resin viscosity and its associated homogenized thermal conductivity.

Simulation allowed for an evaluation of these factors separately, proving that they can have a negative impact on processability. In fact, by increasing the thermal conductivity, heat flows too fast, and the viscosity, already impacted by the presence of charges, is also impacted by the quick temperature decrease, both of these phenomena making consolidation (the degree of intimate contact) difficult. We also proved that the impact depends strongly on the prepreg surface, the effect being much more noticeable when its initial roughness increases.

This study leads the way to different research axes: (i) validating main numerical outputs and (ii) properly defining process windows that should take into account the prepreg surface to better adapt process parameters (heating power and tape deposition speed), among other possibilities.

## Figures and Tables

**Table 1:** Surface roughness parameters according to ISO 4287 (values correspond to the micro- roughness [4]).

Surface	$R_{a}(\mu$m)	$R_{t}(\mu$m)	$R_{s}(\mu$m)
Surface 1	0.8	10.5	103
Surface 2	2.1	25.3	13

**Table 2:** Simulation parameters: specific heat Cp; power index *n* [22]; air and composite conductivities $K_{a}$ and $K_{m}$, the last associated to the plane perpendicular to the fiber direction; thermal exchange coefficients $h_{air}$, $h_{mould}$, and $h_{roller}$ between the composite and respectively the air, mould, and roller; the applied force F; the laying velocity $V_{laser}$; the applied laser power $P_{laser}$; the number of plies *m*; the air and mould temperatures $T_{amb}$ and $T_{mould}$ respectively; and finally the metallic particles conductivity $K_{p}$.

Parameters of Simulation			
ρCp	2.2 × 10${}^{6}$	F	600 N
*n*	0.65	$K_{m}$	0.5 W/(m K)
$K_{a}$	0.024 W/(mK)	$h_{c}$	4000 K m${}^{2}$/W
$h_{air}$	10 K m${}^{2}$/W	$h_{mould}$	2500 K m${}^{2}$/W
$h_{roller}$	2000 K m${}^{2}$/W	$V_{laser}$	0.1 m/s
*m*	6	$T_{amb}$	25 ${}^{\circ }$C
$T_{Mould}$	25 ${}^{\circ }$C	$P_{laser}$	720 W
$K_{p}$	60 W/(m K)		
